# Procalcitonin, MR-Proadrenomedullin, and Cytokines Measurement in Sepsis Diagnosis: Advantages from Test Combination

**DOI:** 10.1155/2015/951532

**Published:** 2015-11-09

**Authors:** Silvia Angeletti, Giordano Dicuonzo, Marta Fioravanti, Marina De Cesaris, Marta Fogolari, Alessandra Lo Presti, Massimo Ciccozzi, Lucia De Florio

**Affiliations:** ^1^Clinical Pathology and Microbiology Laboratory, University Hospital Campus Bio-Medico of Rome, 00128 Rome, Italy; ^2^Department of Infectious, Parasitic, and Immune-Mediated Diseases, Epidemiology Unit, Reference Centre on Phylogeny, Molecular Epidemiology, and Microbial Evolution (FEMEM), National Institute of Health, 00161 Rome, Italy

## Abstract

*Background*. Elevated cytokines levels correlate with sepsis severity and mortality but their role in the diagnosis is controversial, whereas Procalcitonin (PCT) has been largely used. Recently, the mid-regional proadrenomedullin (MR-proADM) has been combined with PCT for diagnosis optimization. In this study the combined measurement of PCT, MR-proADM, and cytokines in patients with sepsis was evaluated. *Methods*. One hundred and four septic patients and 101 controls were enrolled. Receiver operating characteristic (ROC) analysis and multiple logistic regression were used to evaluate applicant markers for sepsis diagnosis. Markers with best Odds Ratio (OR) were combined, and the posttest probability and a composite score were computed. *Results*. Based upon ROC curves analysis, PCT, MR-proADM, IL-6, IL-10, TNF-*α*, and MCP-1 were considered applicant for sepsis diagnosis. Among these PCT, MR-proADM , IL-6, and TNF-*α* showed the best OR. A better posttest probability was found with the combination of PCT with MR-proADM and PCT with IL-6 or TNF-*α* compared to the single marker. A composite score of PCT, MR-proADM, and TNF-*α* showed the best ROC curve in the early diagnosis of sepsis. *Conclusion*. The combination of PCT with other markers should expedite diagnosis and treatment of sepsis optimizing clinical management.

## 1. Introduction

Sepsis is the tenth leading cause of death in the United States and represents 6% of overall death from 1999 to 2005 [1, 2].

Despite new advances in the treatment and prevention of infectious diseases, the incidence of sepsis is increasing [3].

The mortality rate of severe sepsis ranges from 25% to 70% when complicated by shock and multiple organ failure [4, 5]. The incidence of sepsis and septic shock has increased significantly over the past two decades with high economic cost [6].

Sepsis is commonly defined as the presence of infection in conjunction with the systemic inflammatory response syndrome (SIRS); severe sepsis is commonly defined as sepsis complicated by organ dysfunction; and septic shock is commonly defined as sepsis-induced acute circulatory failure characterized by persistent arterial hypotension despite adequate volume resuscitation and not being explained by other causes [7, 8].

SIRS is mediated by innate immune cells, including neutrophils, monocytes, and macrophages. A production of proinflammatory cytokines and chemokines including tumor necrosis factor-alpha (TNF-*α*), IL-6, and IL-8 normally triggers beneficial host innate immune responses to limit the infection and the consequent tissue damage. However, in sepsis, the excessive and prolonged production of these cytokines can produce exaggerated inflammatory responses which is more dangerous than the original infection. This is what happens in severe sepsis, where the excessive production of proinflammatory cytokines causes tissue injury and lethal multiple organ failure [9, 10].

Elevated proinflammatory cytokine levels directly correlate with severity and mortality in human sepsis. Proinflammatory cytokines have a defined role in the pathophysiology of sepsis. In fact, these cytokines contribute to the development of an acute phase response with fever, leukocytosis, alterations of metabolism, and activation of the complement and coagulation cascades. Consequently, persistent elevated levels of these cytokines result in a variety of pathologic reactions leading to induction of hypotension and shock [11].

Some studies have been developed to evaluate the role of cytokine profiles measurement in sepsis diagnosis and prognosis but their diagnostic role is controversial [12–16].

Procalcitonin (PCT) is a polypeptide that has demonstrated the highest reliability in the early diagnosis of sepsis, severe sepsis, or septic shock compared to other plasma biomarkers or clinical data alone [17]. Moreover, PCT has been advocated also to clarify the bacterial origin of some localized infections [18, 19]. The mid-regional proadrenomedullin (MR-proADM) has been shown to play a decisive role in both the induction of hyperdynamic circulation during the early stages of sepsis and the progression to septic shock [20–22] and, recently, it has been reported that MR-proADM differentiates sepsis from noninfectious SIRS with high specificity. Moreover, dosing simultaneously MR-proADM and PCT in septic patients increases the posttest diagnostic probabilities compared to the independent determination of individual markers [23, 24].

The present retrospective study was performed to evaluate the combined measurement of PCT, MR-proADM, and cytokines in patients with sepsis or severe sepsis and septic shock and establish which cytokines are prevalent and may contribute to the early diagnosis of sepsis alone or in combination with other markers. The prognostic value of PCT, MR-proADM, and the most significant cytokines was also evaluated comparing survivor and nonsurvivor patients.

## 2. Materials and Methods

### 2.1. Patients

The retrospective study was performed on 104 plasma samples from consecutive sepsis or severe sepsis and septic shock patients admitted during one year to various medical and surgical units at the University Hospital Campus Bio-Medico of Rome, Italy. Criteria for sepsis definition were the presence of SIRS and a positive blood culture [8]. Each blood culture comprised three sets (time 0, time 30, and time 60) of one aerobic and one anaerobic broth bottles (BACTEC Plus Aerobic/F and BACTEC Plus Anaerobic/F, Becton Dickinson, Franklin Lakes, NJ, USA) per patient drawn during 1-hour period from cases of clinically suspected bloodstream infection. Blood culture vials were incubated in the BACTEC 9240 automated system (Becton Dickinson).

Patients were classified as sepsis patients only if there was a culture-based laboratory confirmed diagnosis of bloodstream infection.

Patients were classified according to clinical signs into sepsis and severe sepsis/septic shock.

APACHE II and SOFA scores were computed. APACHE II scores in sepsis, severe sepsis/septic shock, and SIRS patients were calculated by Medscape, APACHE II scoring system calculator [25]. The SOFA score has been calculated only in sepsis and severe sepsis/septic shock patients to better define the severity of the sepsis [26, 27].

Fifty plasma samples obtained as unused amounts remaining from routine sampling in healthy individuals and 50 plasma samples from patients with SIRS but with negative blood culture were similarly studied. Patients were classified at the time of blood collection as having SIRS, sepsis, severe sepsis, or septic shock. Patients and controls' characteristics are summarized in Table 1.

In order to limit the extension of the results severe sepsis and septic shock patients were grouped together for the analysis.

The study was approved by the Ethic Committee of the University Hospital Campus Bio-Medico, Rome, Italy.

### 2.2. Blood Cultures Collection and Processing

Blood specimens from adult patients were systematically collected in BACTEC bottles containing anaerobic (BACTEC anaerobic (BAA)) or aerobic (BACTEC aerobic (BA)) broth and resins. Blood culture bottles (BC) were incubated in BACTEC FX instrument (Becton Dickinson, Meylan, France) immediately upon arrival in the laboratory. After the check-in blood cultures set were continuously incubated in the BACTEC FX instrument (Becton Dickinson, Meylan, France) for a maximum of 5 days or until they became positive for bacterial growth. BC samples that turned positive were immediately processed for Gram staining and cultivated. Bacterial identification was performed by MALDI-TOF, as previously described [28].

### 2.3. PCT and MR-proADM Plasma Measurement

PCT and MR-proADM plasma concentrations were measured by an automated Kryptor analyzer, using a time-resolved amplified cryptate emission (TRACE) technology assay (Kryptor PCT; Brahms AG; Hennigsdorf, Germany), with commercially available immunoluminometric assays (Brahms) [29].

### 2.4. Cytokines Determination

Twelve cytokines, IL-1*α*, IL-1*β*, IL-2, IL-4, IL-6, IL-8, IL-10, VEGF (vascular endothelial growth factor-*α*), IFN-*γ* (interferon gamma), EGF (epidermal growth factor), MCP-1, and TNF-*α*, were simultaneously measured with the cytokine biochip array using the semiautomated Evidence Investigator (Randox Laboratories Ltd., Crumlin, Co. Antrim, United Kingdom) [30].

### 2.5. Statistical Analysis

Data have been analysed using MedCalc 11.6.1.0 statistical package (MedCalc Software, Mariakerke, Belgium). Plasma levels of PCT, MR-proADM, and cytokines were log-transformed to achieve a normal distribution. The normal distribution of each marker concentration was tested by Kolmogorov-Smirnov test. PCT, MR-proADM, and cytokines in healthy individuals and patients with SIRS and sepsis were compared using Mann-Whitney's test. Multiple logistic regression analysis (stepwise method) using sepsis versus PCT, MR-proADM, IL-6, IL-10, TNF-*α*, and MCP-1 was performed and Odds Ratio (OR) was computed. For OR calculation variables were retained for *p* < 0.05 and removed for *p* > 0.1.

Receiver operating characteristic (ROC) analysis was performed among independent variables associated with sepsis to define the cutoff point for plasma PCT, MR-proADM, and cytokines and their diagnostic accuracy to predict sepsis. ROC curves and areas under the curve (AUCs) were calculated for all markers and compared in sepsis from different pathogens (Gram-positive sepsis, Gram-negative sepsis, and yeast sepsis) versus SIRS patients [31].

Pretest odds, posttest odds, and the consequent posttest probability have been computed to investigate whether combination of PCT, MR-proADM, and cytokines improves posttest probability. Likelihood ratios were used as these tests are not prone to bias due to prevalence rates [32].

### 2.6. Composite Score Calculation Derived from the Combination of the Most Significant Markers: PCT, MR-proADM, and TNF-*α*

Different scores were assigned to PCT, MR-proADM, and TNF-*α* values to calculate a composite score for each of the septic patients, as described in Table 10. Multiple logistic regression analysis, using sepsis as dependent variable and the composite score as independent variable, and ROC analysis were performed to test the accuracy of the composite score in sepsis diagnosis and prognosis. 

## 3. Results

### 3.1. Patients and Controls' Characteristics

The mean age of the 104 sepsis patients (59 men and 45 women) included in the present study was 66 ± 12 years (Table 1). The principal comorbidities of patients with sepsis and the sources of bacteremia are summarized in Table 1.

The most frequently isolated Gram-positive pathogen in sepsis was* S. aureus*; instead, in severe sepsis/septic shock, it was* E. faecalis*. Between Gram-negative blood cultures the most frequent pathogen isolated in sepsis was* E. coli*, and in severe sepsis/septic shock it was* P. aeruginosa*. In sepsis and in severe sepsis/septic shock patients,* C. albicans* was the most frequent isolate in yeast positive cultures. Bacterial isolates from positive blood culture are reported in Table 2.

The comparison of PCT, MR-proADM, and cytokines measured in healthy individuals and patients with SIRS versus patients with sepsis by Mann-Whitney's test is reported in Table 3. The control population consisted of 101 individuals (50 healthy individuals and 51 SIRS patients). The demographic characteristics of the control population included in the study are summarized in Table 1.

The average APACHE II score value was 16.2 and 19.6 in sepsis and severe sepsis/septic shock patients, respectively, corresponding to 24% risk of death for both groups, whereas in SIRS patients, the APACHE II score was 7.1, corresponding to 6% risk of death (Table 1).

The initial average SOFA score value was 3.6 in sepsis patients and 6.3 in severe sepsis/septic shock patients corresponding to a predicted mortality of <33% (Table 1).

### 3.2. PCT, MR-proADM, and Cytokine Levels in Sepsis, SIRS, and Healthy Individuals

Median values, interquartile ranges (25th percentile and 75th percentile), and Mann-Whitney's comparison of PCT, MR-proADM, and 12 cytokines analyzed in all sepsis patients and controls are reported in Table 3.

Furthermore, in all sepsis patients, the median values and interquartile range (25th percentile and 75th percentile) of PCT, MR-proADM, and the 12 cytokines were calculated on the basis of the invading pathogen, Gram-positive, Gram-negative, and yeast, as reported in Table 4.

### 3.3. PCT, MR-proADM, and Cytokine Levels in Severe Sepsis/Septic Shock Patients Compared to Nonsevere Sepsis

**Figure 1:**
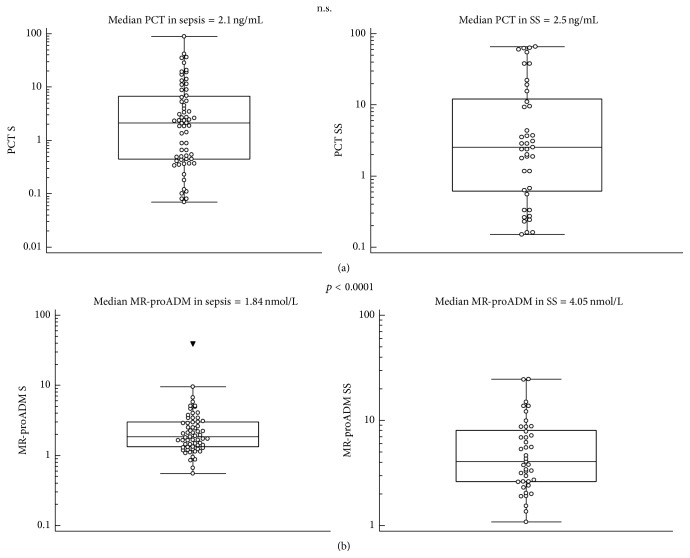
PCT (a) and MR-proADM (b) median values and Mann-Whitney comparison in sepsis (S) and severe sepsis/septic shock (SS) patients.

**Figure 2:**
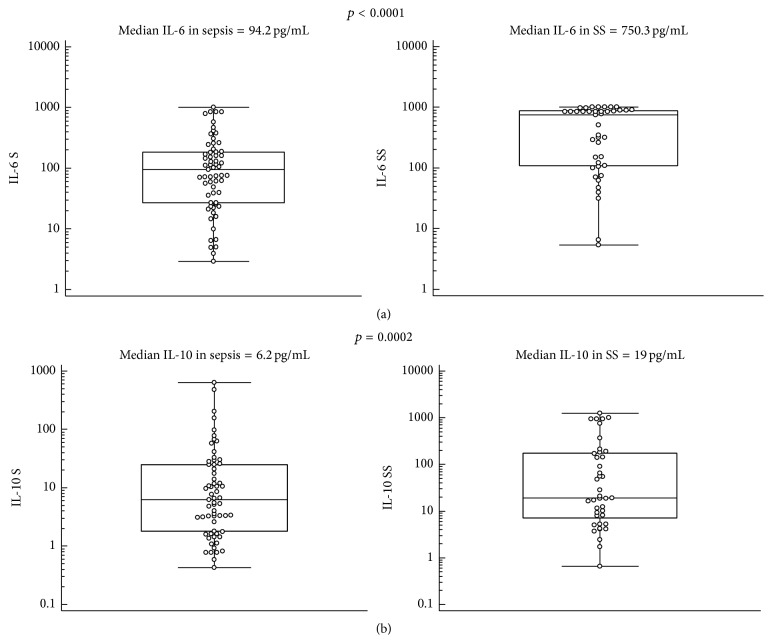
IL-6 (a) and IL-10 (b) median values and Mann-Whitney comparison in sepsis (S) and severe sepsis/septic shock (SS) patients.

**Figure 3:**
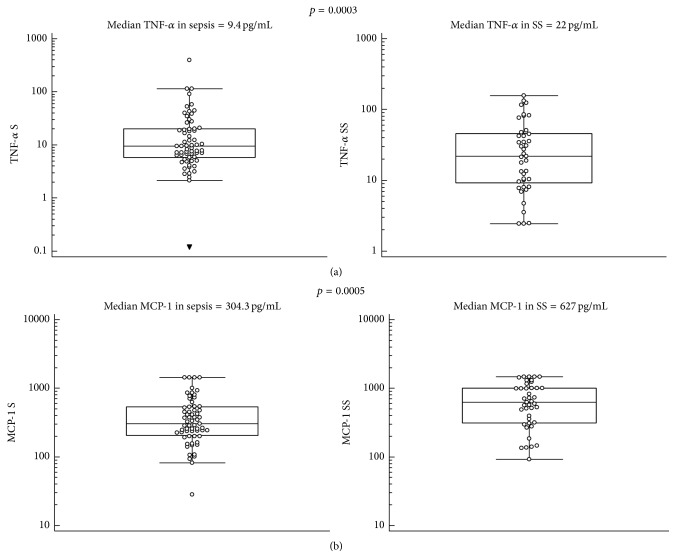
TNF-*α* (a) and MCP-1 (b) median values and Mann-Whitney comparison in sepsis (S) and severe sepsis/septic shock (SS) patients.

The median values and Mann-Whitney's comparison of PCT, MR-proADM, and the most significant cytokines (IL-6, IL-10, TNF-*α*, and MCP-1) in sepsis/septic shock patients in comparison with nonsevere sepsis patients are presented as box plots in Figures 1–3.

### 3.4. ROC Curves and Areas under the Curves (AUCs) Analysis

**Figure 4:**
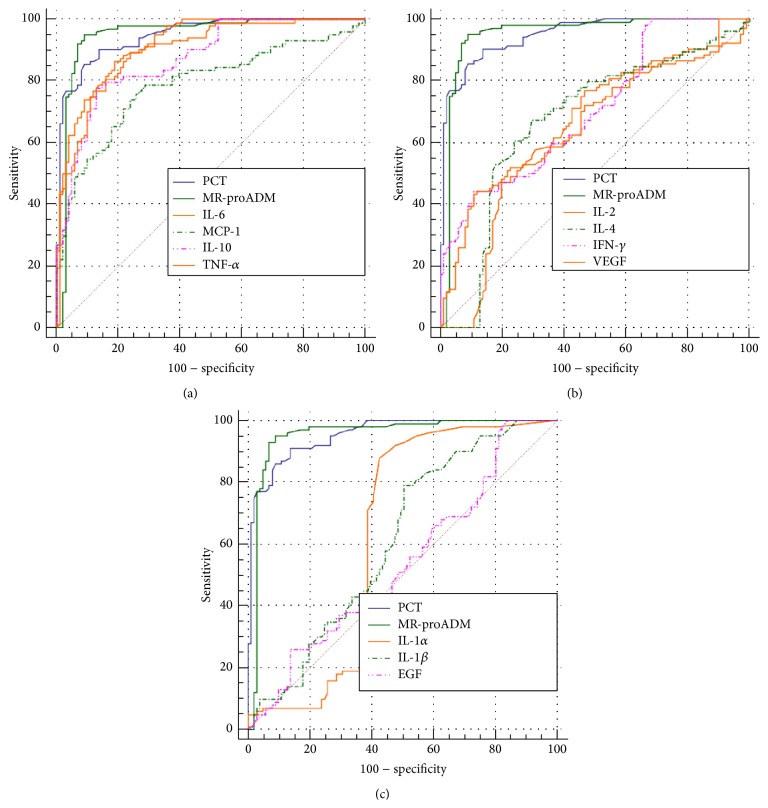
ROC curves comparison in sepsis: (a) PCT versus MR-proADM versus IL-6 versus IL-10 versus TNF-*α* versus MCP-1; (b) PCT versus MR-proADM versus IL-2 versus IL-4 versus INF-*γ* versus VEGF; (c) PCT versus MR-proADM versus IL-1A versus IL-1B versus MCP-1 versus EGF.

In sepsis patients, the AUCs for PCT, MR-proADM, and the 12 cytokines analyzed are reported in Table 4. Based upon ROC curves analysis and AUCs characteristics, PCT, MR-proADM, IL-6, IL-10, TNF-*α*, and MCP-1 were considered applicant for sepsis diagnosis for AUC values ranging from 0.80 to 0.95, as reported in Figure 4 and Table 5. PCT, MR-proADM, and 12 cytokines' AUCs were computed also after dividing sepsis on the basis of the causing pathogen in Gram-negative sepsis, Gram-positive sepsis, and yeast sepsis (Table 4). For PCT the highest AUC value was found in Gram-negative sepsis (0.98 versus 0.93 and 0.91 in Gram-positive sepsis and yeast sepsis, resp.). For MR-proADM the highest value was found in yeast sepsis (0.97 versus 0.94 and 0.96 in Gram-negative sepsis and Gram-positive sepsis, resp.) (Table 3).

In Gram-negative sepsis IL-6 and TNF-*α* showed the best AUC values (both 0.93); in Gram-positive sepsis the best AUC was found for IL-6 (0.91); in yeast sepsis IL-6, IL-8, and IL-10 showed the highest AUC values (0.92 for all) (Table 4).

**Figure 5:**
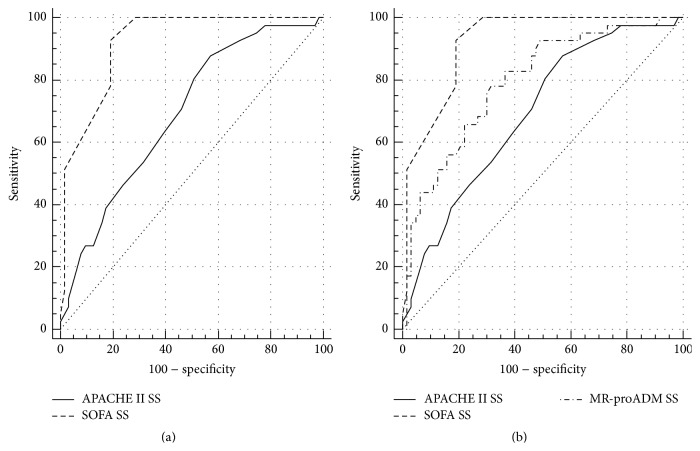
(a) ROC curve comparison of APACHE II and SOFA scores in sepsis (S) and severe sepsis/septic shock (SS) differentiation. (b) ROC curve comparison of APACHE II and SOFA scores and MR-proADM in sepsis (S) and severe sepsis/septic shock (SS) differentiation.

At the ROC analysis, APACHE II resulted to be less accurate in differentiating severe sepsis/septic shock patients from nonsevere sepsis (AUC = 0.69) than SOFA score (AUC = 0.91), whereas MR-proADM had an intermediate accuracy (AUC = 0.79) between APACHE II and SOFA scores, as shown in Figure 5 and Table 6.

### 3.5. Multiple Logistic Regression Analysis

Multiple logistic regression analysis using sepsis as dependent variable and PCT, MR-proADM, and cytokines as independent variables is reported in Table 7. The OR values showed that patients with MR-proADM > 1 nmol/L have about twenty-five times the probability to be affected by sepsis compared to control population (patients with SIRS and healthy subjects), and this high value is confirmed also comparing sepsis patients only with SIRS (twenty-three times compared to SIRS patients) (Table 7). PCT values > 0.5 ng/mL mean the probability to have sepsis twelve times more than control population (patients with SIRS and healthy subjects) and these data are confirmed also comparing sepsis patients only with SIRS (9 times compared to SIRS patients) (Table 7). The only significant OR of having sepsis between different cytokines has been found in IL-6 and TNF-*α* for values > 16 pg/mL and > 5 pg/mL, respectively, as described in Table 5. Interestingly, the only cytokine which in the logistic regression analysis confirms its significance also after comparison of sepsis only with SIRS patients is TNF-*α* (Table 7). Septic patients with APACHE II score > 12 or SOFA score values > 4 have about 4.5 and 60 times, respectively, the probability to be affected by severe sepsis/septic shock.

### 3.6. Combined PCT, MR-proADM, and Cytokines Measurement for Sepsis Diagnosis

Posttest probability analysis was performed to define the diagnostic value in sepsis derived from the use of multiple markers. Posttest probability results are reported in Table 8.

PCT has the highest posttest probability (0.978) when used as single marker and the combination with one or two other markers resulted in higher value of posttest probability (Table 8). The best combination was PCT with MR-proADM leading to a posttest probability of 0.999. The combination of PCT with IL-6 and TNF-*α*, the only cytokines showing significant OR, gave analogous results with posttest probability of 0.995 and 0.997, respectively. To obtain these high values of posttest probability PCT has to be present in the combination. If PCT is excluded in order to reach similar value of posttest probability the combination of MR-proADM, IL-6, and TNF-*α* is necessary (Table 8). When stratified into Gram-positive sepsis, Gram-negative sepsis, and yeast sepsis, this trend is still evident (Table 8).

### 3.7. PCT, MR-proADM, IL-6, IL-10, and TNF-*α*: Survivors and Nonsurvivors

Analysis of PCT, MR-proADM, IL-6, IL-10, and TNF-*α* values in survivor and nonsurvivor patients has been performed. Survivors were 55/63 (87%) and 17/41 (41%) in sepsis and severe sepsis/septic shock patients, respectively (Table 9).

Comparison of PCT, MR-proADM, IL-6, IL-10, and TNF-*α* values in sepsis and severe sepsis/septic shock patients between survivor and nonsurvivor groups is summarized in Table 9.

MR-proADM and IL-10 average values were significantly different (*p* = 0.03 and *p* = 0.04, resp., by Mann-Whitney test for independent samples) between survivors and nonsurvivors in sepsis patients, whereas there was no difference between survivors and nonsurvivors in severe sepsis/septic shock patients (Table 9). PCT in sepsis patients and in severe sepsis/septic shock patients was not different between survivors and nonsurvivors (Table 9).

### 3.8. Composite Score Calculation Derived from the Combination of the Most Significant Markers: PCT, MR-proADM, and TNF-*α*

**Figure 6:**
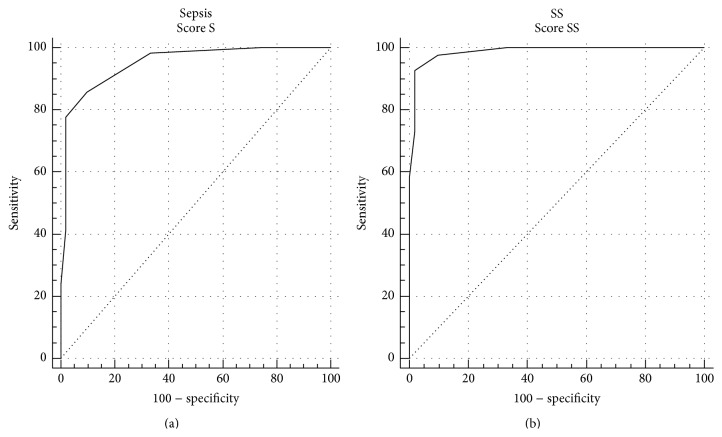
ROC curve of the composite score computed by PCT, MR-proADM, and TNF-*α* combination in sepsis (S) versus SIRS (a) and severe sepsis/septic shock (SS) versus SIRS (b).

For each of the septic patients a composite score was calculated using PCT, MR-proADM, and TNF-*α* scores (Table 10). In nonsevere sepsis the median composite score value was 5, whereas in severe sepsis/septic shock patients it was 8: the difference between the two groups of patients was statistically significant (*p* < 0.0001 with Mann-Whitney's test). In SIRS the median value was 3 and the difference with sepsis or severe sepsis/septic shock was statistically significant (*p* < 0.0001 at Mann-Whitney's test). ROC curve analysis was performed to establish the accuracy of the composite score in sepsis. The composite score showed an AUC of 0.95 and 0.99 in sepsis and severe sepsis/septic shock patients, respectively. ROC curves are reported in Figure 6. The OR values calculated by the logistic regression analysis showed that patients with a composite score > 5 have about 175 times the probability to be affected by sepsis compared to SIRS. Moreover, septic patients with a composite score > 5 have a probability 4 times higher to develop a severe sepsis.

## 4. Discussion

Sepsis is a leading cause of mortality in hospitalized patients and particularly in critically ill patients in Intensive Care Units (ICU). An aggressive diagnostic and therapeutic approach is needed for this syndrome and it is mandatory to distinguish sepsis from SIRS. PCT was regarded some years ago as a great upgrade in the diagnosis of sepsis but it cannot be considered as the only diagnostic marker for sepsis and it seems useful to look for other markers. MR-proADM plays a decisive role in both the induction of hyperdynamic circulation during the early stages of sepsis and the progression to septic shock [20–22].

Recently, it has been shown that MR-proADM differentiates sepsis from noninfectious SIRS with high specificity and dosing simultaneously MR-proADM and PCT in septic patients increases the posttest diagnostic probabilities compared to the independent determination of individual markers [23, 24].

In this study, the combined measurement of PCT, MR-proADM, and cytokines in patients with sepsis was evaluated to establish whether an advantage for sepsis diagnosis derives from the combination of PCT with other markers.

As shown in Table 7, the best OR values for sepsis diagnosis were obtained using PCT, MR-proADM, IL-6, and TNF-*α*. The combination of PCT with MR-proADM gave the best posttest probability (0.999), but also the combination of PCT with IL-6 or TNF-*α*, the only cytokines showing significant OR, gave analogous results with posttest probability of 0.995 and 0.997, respectively (Table 8). To obtain these high values of posttest probability PCT has to be present in the combination. The exclusion of PCT from the combination requires the association of at least three markers to obtain similar results (Table 8). When stratified into Gram-positive sepsis, Gram-negative sepsis, and yeast sepsis, this trend is still evident (Table 8).

Theoretically, proinflammatory cytokines (IL-6 and TNF-*α*) and anti-inflammatory cytokines (IL-4 and IL-10) are increased during sepsis even if IL-4 is often found at low levels probably owing to its short half-life in plasma (5–19 minutes) [33, 34]. IL-6 and IL-10 are significantly increased in most septic patients, while TNF-*α* and IFN-*γ* are mostly increased in patients with severe disease. IL-6 is strongly related to hemodynamic disorder, while TNF-*α* and IFN-*γ* are related to disease severity [35].

Gram-negative sepsis showed the highest AUC value for proinflammatory cytokines such as IL-6 and TNF-*α* compared to Gram-positive sepsis and yeast sepsis, which at least in part could be explained by the interaction of lipopolysaccharide and TLR4 [36]. In yeast sepsis the cytokine IL-10 showed the best AUC values compared to Gram-negative sepsis and Gram-positive sepsis. During fungal infection, macrophages and dendritic cells (DCs) recognize evolutionary conserved components from the fungal cell wall through their pattern recognition receptors (PRRs) which triggers a series of signaling cascades leading to activation of various transcription factors. Yeast interacts directly with Toll-like receptors (TLRs) playing a central role in immunity to fungal pathogens and a receptor-mediated response including the release of cytokines such as IL-10 is induced [37].

Data from this study confirmed the important diagnostic role of PCT in sepsis diagnosis, being the only marker that alone reaches a significant posttest probability (0.978), as well as the usefulness from the combination of PCT and MR-proADM leading to a posttest probability near to 100%. Furthermore, this study found that, among a panel of 12 cytokines, IL-6 and TNF-*α* seem to be the only cytokines with a relevant role in the diagnosis of sepsis and that their association with PCT leads to a posttest probability comparable to that achieved by PCT and MR-proADM combination.

The combination of PCT with other markers should contribute to a more specific diagnosis and prompt treatment of patients as well as to the evaluation of the infection severity and death risk, with MR-proADM and TNF-*α* being recognized as markers of disease severity and death risk [14, 23, 37, 38].

MR-proADM and IL-10 were significantly different between survivor and nonsurvivor patients. Furthermore, MR-proADM showed a good accuracy in differentiating severe sepsis/septic shock patients from nonsevere sepsis, which was higher than APACHE II score but lower than SOFA score. These data confirm the role of MR-proADM as a marker of infection severity and death risk as previously reported [18] and underline also the possible role of IL-10 as a prognostic marker which could be explained by its anti-inflammatory action [39].

A composite score was also calculated combining PCT, MR-proADM, and TNF-*α*, the markers that resulted to be the most significant in the logistic regression analysis. The combined use of the three markers in a composite score showed a very high degree of accuracy in the diagnosis and prognosis of sepsis.

## 5. Conclusions

Data from this study should contribute to elucidating the role of PCT and other markers such as MR-proADM and inflammatory cytokines in sepsis diagnosis and prognosis. It also illustrated the role of the combined use of the three markers, PCT, MR-proADM, and TNF-*α*, leading to their rational use in combination which should expedite a timely diagnosis and treatment of sepsis and add information on the prognosis of septic patients to optimize the clinical management.

## Figures and Tables

**Table 1:** Demographic characteristics of the sepsis patients, SIRS patients, and the healthy individuals included in the study population.

Sepsis patients	

Number of patients	104
Mean age	66 ± 12
Male	59
Female	45
Sepsis	63
Gram-positive	26
Gram-negative	34
Yeast	3
Severe sepsis/septic shock	41
Gram-positive	15
Gram- negative	19
Yeast	7
APACHE II score	
Sepsis 16.2 (surv.: 15.4; nonsurv.: 20.2)	
Severe sepsis 19.6 (surv.: 18.5; nonsurv.: 20.7)	
SOFA score	
Sepsis 3.6 (surv.: 2.6; nonsurv.: 5.2)	
Severe sepsis 6.3 (surv.: 5; nonsurv.: 7)	
With comorbidities	
Diabetes	8
Malignancy	48
Hematological malignancies	13
Cardiovascular disease	10
Gastrointestinal disease	6
Autoimmune disease	4
Renal failure	5
Others	12
Site of primary infection	
Pneumoniae	9
Intra-abdominal	29
Urinary tract infection	20
CVC colonization	14
Soft tissue	11
Unknown	21

SIRS patients	

Number of patients	51
Mean age	60 ± 10
Male	27
Female	24
APACHE II score	
7.9 (surv.: 7.1; nonsurv.: 9.6)	
With comorbidities	
Diabetes	2
Malignancy	15
Hematological malignancies	36
Cardiovascular disease	2
Gastrointestinal disease	4
Autoimmune disease	2
Hepatic failure	1
Others	4

Healthy controls	

Number of patients	50
Mean age	65 ± 12
Male	35
Female	15

surv.: survivor; nonsurv.: nonsurvivor.

**Table 2:** Bacterial isolates from positive blood culture.

	Gram-positive	Gram-negative	Yeast
Sepsis	14 *S. aureus*	17 *E. coli*	2 *C. albicans*
	4 *S. epidermidis*	5 *K. pneumoniae*	1 *C. parapsilosis*
	2 *E. faecalis*	2 *P. aeruginosa*	
	2 *S. hominis*	2 *P. mirabilis*	
	1 *S. hyicus*	2 *B. fragilis*	
	1 *S. haemolyticus*	1 *E. aerogenes*	
	1 *E. durans*	1 *K. oxytoca*	
	1 *S. sanguis*	1 *S. maltophilia*	
		1 *C. freundii*	
		1 *A. naeslundii*	
		1 *E. cloacae*	

Total number: 63	26	34	3

Severe sepsis/septic shock	5 *E. faecalis *	7 *P. aeruginosa*	3 *C. albicans*
	3 *S. aureus*	5 *E. coli*	2 *C. parapsilosis*
	3 *S. epidermidis*	5 *K. pneumoniae*	1 *C. glabrata*
	2* E. faecium*	1 *K. oxytoca*	1 *C. tropicalis*
	1 *S. haemolyticus*	1 *B. fragilis*	
	1 *S. anginosus*		

Total number: 41	15	19	7

**Table 3:** Median value, interquartile range (IR) (25th percentile and 75th percentile), and Mann-Whitney comparison of PCT, MR-proADM, and cytokines in sepsis, SIRS, and healthy individuals.

Variable	Sepsis median (IR)	SIRS median (IR)	Healthy median (IR)	Sepsis versus SIRS Mann-Whitney's test	Sepsis versus healthy Mann-Whitney's test
PCT ng/mL	2.35 (0.47–9.08)	0.12 (0.06–0.25)	0.04 (0.03–0.07)	<0.0001	<0.0001

MR-proADM nmol/L	2.55 (1.59–4.60)	0.77 (0.62–0.86)	0.42 (0.28–0.63)	<0.0001	<0.0001

IL-2 pg/mL	5.02 (2.98–6.97)	5.80 (4.90–7.80)	8.79 (7.15–11.58)	0.047	0.0003
IL-4 pg/mL	1.64 (0.44–2.50)	2.10 (1.53–2.53)	3.65 (3.21–5.08)	n.s.	<0.0001
IL-6 pg/mL	137.1 (52.30–654.78)	16.3 (3.05–52.30)	1.24 (0.73–1.75)	<0.0001	<0.0001
IL-8 pg/mL	21.07 (6.41–69.17)	5.31 (2.59–12.70)	3.49 (2.81–6.01)	<0.0001	<0.0001
IL-10 pg/mL	10.43 (3.31–51.60)	1.51 (0.10–2.85)	0.03 (0.01–1.77)	<0.0001	<0.0001
VEGF pg/mL	64.26 (29.52–134.09)	47.20 (20.08–73.49)	28.20 (46.00–74.20)	0.021	<0.0001
IFN-*γ* pg/mL	3.78 (1.67–13.03)	2.50 (1.26–4.06)	0.09 (0.02–1.65)	0.0048	<0.0001
TNF-*α* pg/mL	11.69 (6.83–34.59)	1.94 (1.50–3.78)	2.86 (0.06–3.67)	<0.0001	<0.0001
IL-1*α* pg/mL	0.21 (0.11–0.46)	0.05 (0.02–0.63)	0.09 (0.02–1.65)	n.s.	0.0003
IL-1*β* pg/mL	1.25 (0.80–2.86)	0.09 (0.03–1.44)	1.28 (0.08–3.22)	0.0003	n.s.
MCP-1 pg/mL	401.4 (401.42–752.63)	210.76 (14.95–333.08)	158.50 (121.40–196.40)	<0.0001	<0.0001
EGF pg/mL	3.65 (1.54–13.47)	2.88 (0.32–6.90)	4.09 (1.78–11.21)	n.s.	n.s.

**Table 4:** Median value, interquartile range (IR) (25th percentile and 75th percentile), and areas under the curves (AUCs) values in Gram-negative sepsis, Gram-positive sepsis, and yeast sepsis.

	Median	Interquartile range	AUC
		(25th percentile and 75th percentile)	
*Gram-negative sepsis*			

PCT ng/mL	3.5	1.23–18.43	0.98

MR-proADM nmol/L	2.51	1.40–5.04	0.94

IL-2 pg/mL	5.23	7.34–10.35	0.62
IL-4 pg/mL	1.62	0.43–2.32	0.67
IL-6 pg/mL	168.3	61.00–833.81	0.93
IL-8 pg/mL	31.24	9.56–114.67	0.83
IL-10 pg/mL	18.87	3.33–74.26	0.85
VEGF pg/mL	66.49	33.11–146.39	0.70
IFN-*γ* pg/mL	6.24	2.49–16.10	0.76
TNF-*α* pg/mL	21.33	7.61–46.91	0.93
IL-1*α* pg/mL	0.20	0.10–0.50	0.62
IL-1*β* pg/mL	1.27	0.91–3.43	0.63
MCP-1 pg/mL	521.33	256.57–990.00	0.85
EGF pg/mL	4.45	2.17–14.05	0.58

*Gram-positive sepsis*			

PCT ng/mL	1.09	0.33–2.62	0.93

MR-proADM nmol/L	2.14	1.69–3.43	0.96

IL-2 pg/mL	4.77	1.41–6.22	0.67
IL-4 pg/mL	0.82	0.35–2.41	0.70
IL-6 pg/mL	103.80	48.00–287.21	0.91
IL-8 pg/mL	10.26	5.29–34.53	0.71
IL-10 pg/mL	5.75	2.34–11.60	0.85
VEGF pg/mL	70.34	29.52–122.10	0.67
IFN-*γ* pg/mL	2.49	0.32–4.42	0.59
TNF-*α* pg/mL	7.73	5.86–11.81	0.87
IL-1*α* pg/mL	0.19	0.11–0.36	0.61
IL-1*β* pg/mL	0.94	0.09–1.83	0.55
MCP-1 pg/mL	292.37	191.76–492.59	0.74
EGF pg/mL	3.39	1.44–12.59	0.53

*Yeast sepsis*			

PCT ng/mL	1.38	0.24–3.68	0.91

MR-proADM nmol/L	4.74	2.40–8.71	0.97

IL-2 pg/mL	5.76	4.19–45.27	0.51
IL-4 pg/mL	2.15	1.68–3.91	0.56
IL-6 pg/mL	354.68	70.88–850.00	0.92
IL-8 pg/mL	54.96	19.59–87.76	0.92
IL-10 pg/mL	30.57	10.39–155.20	0.92
VEGF pg/mL	48.38	12.74–103.49	0.57
IFN-*γ* pg/mL	14.59	5.54–37.06	0.85
TNF-*α* pg/mL	21.52	9.56–42.00	0.89
IL-1*α* pg/mL	0.34	0.28–0.62	0.63
IL-1*β* pg/mL	2.25	1.23–13.73	0.75
MCP-1 pg/mL	530.29	355.83–857.90	0.82
EGF pg/mL	2.63	1.64–13.46	0.55

**Table 5:** Receiver operating characteristic (ROC) curves: areas under the curves (AUCs) characteristics and cutoff values for PCT, MR-proADM, and cytokines in sepsis patients.

Markers	AUC	Sensitivity %	Specificity %	LR+	Cutoff value
PCT	0.91	75	96	19.13	0.5

MR-proADM	0.95	92	92	11.77	1.0

IL-6	0.92	89	76	3.70	16
IL-10	0.87	78	87	6.05	3
TNF-*α*	0.90	86	81	4.60	5
IL-8	0.78	70	73	2.63	9
MCP-1	0.80	74	76	3.12	240
VEGF	0.67	44	89	3.97	89
IFN-*γ*	0.70	44	89	4.06	5
IL-1*α*	0.63	88	57	2.07	0.08
IL-1*β*	0.61	79	49	1.56	0.33
EGF	0.54	97	19	1.20	0.22
IL-2	0.64	77	53	1.65	<7
IL-4	0.67	67	70	2.27	<2

**Table 6:** Receiver operating characteristic (ROC) curves: areas under the curves (AUCs) characteristics and cutoff values for APACHE II and SOFA scores in all sepsis (sepsis + SS), sepsis, and SS patients.

	APACHE II				SOFA
	All sepsis versus SIRS	Sepsis versus SIRS	SS versus SIRS	SS versus sepsis	SS versus sepsis
AUC	0.93	0.91	0.98	0.82	0.91
Sensitivity %	83	75	95	95	91
Specificity %	99	99	99	58	80
LR+	3.64	3.31	4.15	2.31	4.55
Cutoff value	12	12	12	12	4

SS, severe sepsis/septic shock patients; SIRS, systemic inflammatory response syndrome.

**Table 7:** Multiple logistic regression analysis: sepsis versus PCT, MR-proADM, IL-6, IL-10, TNF-*α*, and MCP-1 (variables were included if *p* < 0.05 and removed if *p* > 0.1). (A) The group of sepsis patients (104 subjects) were compared with SIRS patients and healthy individuals (101 subjects). (B) Sepsis patients (104 subjects) were compared only with SIRS patients (51 subjects).

Independent variable	OR	95% CI	*p* value
(A) Sepsis: dependent variable			
PCT	12.6136	1.7315 to 91.8885	0.0124
MR-proADM	25.8435	6.9528 to 96.0593	<0.0001
IL-6	7.2262	2.0087 to 25.9954	0.0025
TNF-*α*	4.8752	1.3204 to 18.0004	0.0175

(B) Sepsis: dependent variable			
PCT	9.4602	1.5411 to 58.0723	0.0152
MR-proADM	23.3066	5.9188 to 91.7756	<0.0001
TNF-*α*	10.4546	2.7075 to 40.3693	0.0007

**Table 8:** Posttest probability analysis used to define the diagnostic value derived from the combined use of PCT, MR-proADM, IL-6, and TNF-*α* testing in all sepsis patients and after stratification in Gram-positive sepsis, Gram-negative sepsis, and yeast sepsis.

Markers	All sepsis	Gram-positive	Gram-negative	Yeast
PCT	0.978	0.920	0.960	0.780
ADM	0.930	0.840	0.85	0.763
IL-6	0.810	0.570	0.670	0.250
TNF-*α*	0.843	0.660	0.790	0.310

Markers association	All sepsis	Gram-positive	Gram-negative	Yeast

PCT + ADM	0.999	0.994	0.995	0.970
PCT + IL-6	0.995	0.977	0.990	0.920
ADM + IL-6	0.980	0.95	0.960	0.750
PCT + TNF-*α*	0.997	0.982	0.995	0.970
ADM + TNF-*α*	0.983	0.962	0.975	0.890
ADM + IL-6 + TNF-*α*	0.996	0.988	0.996	0.966
IL-6 + TNF-*α*	0.952	0.861	0.901	0.613

**Table 9:** PCT, MR-proADM, IL-6, IL-10, and TNF-*α* average values in sepsis and severe sepsis/septic shock (SS) patients: comparison between survivor and nonsurvivor.

	Sepsis		*p*	SS		*p*
	GM surv. (*n* = 55)	GM nonsurv. (*n* = 8)		GM surv. (*n* = 17)	GM nonsurv. (*n* = 24)	
PCT ng/mL	1.7	2.4	n.s.	2.6	2.7	n.s.
MR-proADM nmol/L	2.0	2.9	0.03	4.1	4.9	n.s.
IL-6 pg/mL	70	163	n.s.	284	307	n.s.
IL-10 pg/mL	6.1	28.6	0.04	27.9	39.2	n.s.
TNF-*α* pg/mL	10	21	n.s.	27.8	17.2	n.s.

GM, Geometric Mean; surv., survivor; nonsurv., nonsurvivor; *n*, number.

**Table 10:** Scores assigned to PCT, MR-proADM, and TNF-*α* used for the composite score calculation.

Score	PCT (ng/mL)	MR-proADM (nmol/L)	TNF-*α* (pg/mL)
0	<0.05	<0.5	<1.49
1	0.05–049	0.5–0.99	1.5–3.99
2	0.5–1.99	1–2.99	4–9.99
3	2.00–9.00	3–7.99	10–19.99
4	>10	>8.00	>20
